# Factors affecting conspiracy theory endorsement in paranoia

**DOI:** 10.1098/rsos.211555

**Published:** 2022-01-26

**Authors:** A. G. Greenburgh, A. Liefgreen, V. Bell, N. Raihani

**Affiliations:** ^1^ Psychology and Language Sciences, University College London, London, UK; ^2^ Research Department of Clinical, Educational, and Healthy Psychology, University College London, London, UK; ^3^ South London and Maudsley NHS Foundation Trust, London, UK

**Keywords:** conspiracy thinking, paranoia, belief

## Abstract

Paranoia and conspiracy thinking are known to be distinct but correlated constructs, but it is unknown whether certain types of conspiracy thinking are more common in paranoia than others. In a large (*n* = 1000), pre-registered online study we tested if endorsement of items on a new Components of Conspiracy Ideation Questionnaire varied according to whether harm was described as being (a) intentional and (b) self-referential. Our predictions were supported: paranoia was positively associated with endorsement of items on this questionnaire overall and more paranoid individuals were more likely to endorse items describing intentional and self-referential harm. Belief in any item on the Components of Conspiracy Ideation Questionnaire was associated with belief in others and items describing incidental harm and harm to others were found to be more believable overall. Individuals who endorsed conspiracy theory items on the questionnaire were more likely to state that people similar to them would as well, although this effect was not reduced in paranoia, counter to our expectations.

## 1. Introduction

Conspiracy theories have been defined as ‘attempts to explain the hidden causes of significant social and political events and circumstances with claims of secret plots by two or more powerful actors' [1, p. 4] where these actors are often seen as malevolent [1]. Perhaps unsurprisingly, paranoia, the tendency to believe that harm will occur and that it is intended by other people, is associated with belief in conspiracy theories [2–4]. Paranoia exists as a continuum in the population, where many people harbour mild paranoid concerns and a minority of people hold frank persecutory delusions [5–7]. Conspiracy theories vary quite broadly in various properties, including whether any harmful outcome is intended by the malevolent actors (or occurs as an incidental by-product), and whom the target of this harm is (i.e. the general population or a specific individual or group of individuals). Although proneness to paranoid thinking and a tendency to endorse conspiracy theories clearly overlap—and share some underlying putative risk factors—it is not yet clear if the association between paranoia and conspiracy thinking is dependent on certain properties of conspiracy theories themselves. In this pre-registered study, we asked whether belief in conspiracy theories with certain features is more likely to be associated with paranoid thinking.

In addition to paranoia, several other psychological and social factors are associated with conspiracy thinking. These include personality traits, such as the need for certainty and uniqueness [8]; variation in cognition, such as erratic belief updating and attributional and perceptual biases [9–12]; conservative political orientation ([4,13]; but see [14]), low trust in authorities, adverse personal circumstances, inequality, societal crises, polarization and misinformation [15–17]. However, although we know rather a lot about factors predisposing people to conspiracy thinking in general terms, far less attention has been paid to how the themes and content of the conspiracy theories themselves affect endorsement, and whether this varies among individuals. Additionally, although evidence suggests that belief in one conspiracy theory predicts belief in others [18], it is unknown whether this relationship is stronger for conspiracy theories that share certain attributes.

Studies exploring the link between paranoia and conspiracy thinking have tended to calculate associations between paranoia and measures of general conspiracy mindset*.* These measures pose broad statements such as ‘The government is involved in the murder of innocent citizens and/or well-known public figures, and keeps this a secret’ and ‘I think that the official version of events given by authorities very often hides the truth’ [4,19,20]. Because these approaches often involve aggregating responses across multiple items [12] or measuring conspiracy thinking using one item alone [2], they cannot speak to the relationship between paranoia and different features of conspiracy thinking.

Although meta-analytic evidence suggests a moderately strong association between paranoia and conspiracy thinking, (*Fishers Z* = 0.38, [4]), conspiracy thinking does not necessarily stem from an underlying paranoid disposition. Indeed, although they are correlated, a recent study employing a multi-trait, multi-method approach showed that paranoia and conspiracy thinking are distinct constructs: paranoia is more closely related to self-relevant constructs (e.g. personality traits such as introversion and neuroticism) whereas conspiracy thinking shows stronger associations with constructs pertaining to socio-political domains (e.g. low trust in government) [4]. Accordingly, the conspiracy ‘mindset’ has been viewed as a generalized political attitude, or a cognitive schema, without broad clinical relevance [4,21,22]. Given they are distinct but correlated constructs, it is possible that paranoia relates to certain aspects of conspiracy thinking more than others.

The majority of work on paranoia and conspiracy thinking examines the relationship in the population as a whole where the majority of people will not be distressed or disabled by the intensity or intrusiveness of their beliefs. Paranoia can be highly distressing and, at the upper extremity of the paranoia continuum, forms a core part of psychosis [6]. It is clear that conspiracy beliefs are common in people with paranoid delusions [23] and one distinguishing feature may be that, as paranoia becomes more delusional, concerns about conspiracies are more likely to involve the believer rather than simply focusing on ‘significant social and political events' [24]. This suggests that perception of conspiracies and the type of conspiracy may change as paranoia becomes more severe.

Consequently, to study conspiracy thinking in the current study, we sought to understand how different features of conspiracy theories influence how strongly they are endorsed. Namely, we focus on whether the harmful outcome is described as intended and who is said to be affected by it. First, conspiracy theories imply intentional harm to different extents. For example, the conspiracy theory that the government has been taken over by Satanists to facilitate child abuse implies a higher level of intent to harm than the belief that the moon landings were faked. Second, the target of the harm described in conspiracy theories can vary: some conspiracy theories imply society as a whole will be harmed, some name individuals or groups of individuals as the victim(s) of the harmful conspiracy, whereas some believers might hold conspiracy beliefs about themselves personally being targeted. Endorsement of conspiracy theories may vary according to these features.

Further, by decomposing conspiracy theories according to their features, we examine whether different elements of conspiracy theories may drive the association between conspiracy thinking and paranoia. A potential distinguishing factor between paranoia and general conspiracy thinking is that paranoia is largely self-focused ([4] although see [25]). We, therefore, expected paranoia to be associated with increased endorsement of conspiracy theories that describe the believer themselves as the target or victim of a given event. Given that paranoia in the general population is associated with stronger attributions of harmful intent [25–27], and higher levels of perceived intentionality of negative events [28], we also expected paranoia to be associated with stronger belief that the harmful events described in conspiracy theories are *intended* outcomes, rather than incidental side-effects.

It is clear that factors unrelated to the content of conspiracy theories themselves may increase the degree to which people believe in them—one such factor is social influence. Cognitive models emphasize that beliefs are not held simply for the verity or credibility of their claims and content, but that we adapt our beliefs to the social context, where beliefs that are socially rewarded are held more strongly [29]. Therefore, beyond the concrete features of conspiracy theories, conspiracy thinking likely additionally depends on the beliefs of one's in-group. Increasing evidence supports this claim: conspiracy thinking is predicted by social interaction with other conspiracy believers, and marginalization outside of such communities—even to a greater extent than by individual variation in psychological factors such as anger, sadness and anxiety [30]. However, given that paranoia involves social avoidance, isolation and reduced identification with some social groups [31–33] and that social identification with a group leads to conformity of behaviour to the group [34], it may be that the tendency to shape one's conspiracy beliefs to match one's in-group is reduced in paranoia.

We made a number of pre-registered predictions for our experimental study. First, we expected paranoia to be associated with a tendency to endorse conspiracy theories, and particularly with endorsement of self-referential conspiracy theories and where harmful outcomes were described as intentional. Second, we expected that people who endorsed a conspiracy theory of one type would be more likely to endorse other conspiracy theories of that same type: categories of conspiracy thinking would be distinguishable according to the level of intentionality and the target they describe. Finally, we predicted that individuals would be more likely to believe conspiracy theories that they thought others similar to themselves would also believe but that this effect would be reduced in paranoia.

## 2. Method

Full materials, data and code are available at https://osf.io/zx8me/?view_only=d02e5abdf6304fb0885ccf32853934ca. The study design, sample size, exclusion criteria and analyses were pre-registered at https://aspredicted.org/blind.php?x=wa2jh4. We note below where relevant some deviations from the pre-registered analyses.

### 2.1. Participants

This study was carried out in November 2020 and received full ethics approval. All participants were fully informed as to the nature of the study and participation was voluntary. In line with our pre-registration, we recruited 1000 US-based participants from Prolific Academic (www.prolific.ac), the online crowdsourcing platform. In order to recruit participants across a range of conspiracy ideation, we pre-registered that we would aim at initially recruiting 1000 participants to take part in the study, after which we would determine the proportion of the sample who scored over 75/120 in endorsement of the Components of Conspiracy Ideation Questionnaire—an average of 3.15 in response to each conspiracy theory. If the proportion of the sample meeting this condition was less than 7%, we stated we would recruit more participants, until this criterion was met—in accordance with the distribution of paranoid thinking in previous studies [27], with an upper limit of 2000 participants in total. Any participants recruited after the initial 1000 would only have been included in the sample if they scored over 75/120 in the Components of Conspiracy Ideation Questionnaire. However, as 14.5% of our initial sample scored above 75/120, we did not recruit any more than the initial 1000.

The mean age of the sample was 36 (s.d. = 12), with a small male majority (Male = 522, Female = 463, Nonbinary = 13, Other = 1, Prefer not to say = 1). The sample had a mild conservative bias in political orientation (table 1). Participants were paid £2.20 for taking part in this study and could earn a bonus for passing attention checks. All participants completed questionnaires measuring paranoid ideation, conspiracy thinking and social and economic conservatism (described below).

**Table 1.** Summary statistics for main measures.

Questionnaire	range	mean	s.d.
Persecution Subscale, R-GPTS (total)	10–40	15.04	6.99
Reference Subscale, R-GPTS (total)	8–32	13.92	5.73
Components of Conspiracy Ideation Questionnaire (per item)	1–5	2.42	0.75
General Conspiracy Mindset Questionnaire (per item)	0–100	65.2%	19.53
Social and Economic Conservatism Scale (per item)	4–99	56.53	20.17

### 2.2. Measures

#### 2.2.1. Paranoia

All participants completed the Revised Green *et al.* Paranoid Thoughts Scale (R-GPTS) [35]. This scale comprises two subscales that measure ideas of reference and ideas of persecution, respectively. Scores on the persecution subscale of the R-GPTS can range from 0 to 40, and from 0 to 32 on the reference subscale. These two subscales have been validated for separate use and both show high reliability across the spectrum of severity (*α* > 0.90) [35]. A previous study reported the following mean scores on the persecution subscale: 4.53 (s.d. = 6.74) for participants from the general population; 13.7 (s.d. = 13.0) for patients with psychosis; 26.1 (s.d. = 9.46) for participants with persecutory delusions [35]. We used the persecution subscale of the R-GPTS as a proxy for trait paranoia and, in line with our preregistration, reconducted our main regression analyses using the reference subscale.

#### 2.2.2. Components of conspiracy ideation

We designed a novel 24-item questionnaire, which we call the Components of Conspiracy Ideation Questionnaire, to test our main predictions. Scores on the Components of Conspiracy Ideation Questionnaire designed for this study could range from 24 to 120 in total. Each item on the questionnaire was a statement of an explanation of harmful event, and participants indicated the extent to which they endorsed this explanation on a scale of 1–5 (strongly disagree—strongly agree).

The items varied according to three conditions: target (society as a whole, or targeting the respondent), the intentionality of harm (whether the harm was intentional or an incidental by-product of the action described), and the specificity (whether a general scenario was described or if specific details were included). Specificity was only varied within the target = society condition (table 2, and see electronic supplementary material for full questionnaire), as having high specificity in the target = self condition was hard to achieve.

**Table 2.** All conspiracy theory items from one example theme (vaccination) in the Components of Conspiracy Ideation Questionnaire.

type number	1	2	3	4	5	6
intentionality =	intentional	incidental	intentional	incidental	intentional	incidental
target =	self	self	society	society	society	society
specificity =	general	general	general	general	specific	specific
example conspiracy theory item	some of the vaccines I have received have been designed to be harmful to me, but I was unaware of this at the time	some of the vaccines I have received have later been discovered to be harmful, but I have not been officially informed of this	vaccines have been designed to harm the public and most people do not know this	vaccines given to the public have unintended harmful side effects and the public are unaware of this	the MMR (measles, mumps and rubella) vaccine was intentionally designed to give children autism, and the public was unaware of this	the MMR (measles, mumps and rubella) vaccine causes autism in children, but the public has not been officially warned of this

Altogether the Components of Conspiracy Ideation Questionnaire comprised six types of item 1: intentional/self/general, 2: incidental/self/general, 3: intentional/society/general, 4: incidental/society/general, 5: intentional/society/specific, 6: incidental/society/specific. We used four themes for each condition: data privacy, vaccination, international relations, and poisoning. Within each theme, the wording and content in each item were standardized, so that the main variation within each theme depended on the condition (intentionality/target type/specificity). Together, the six types of item, each taking four possible themes, gave 24 questionnaire items overall.

Therefore, the items were designed to systematically vary and isolate components of conspiracy theories in order to investigate the impact these features have on belief. For example, any increased endorsement of intentional items compared to incidental items could be attributed to the variation in the intentionality dimension alone. While many items on the questionnaire were direct conspiracy theories, items in the intentionality = incidental category did not necessarily reflect true conspiracy theories; however, this allowed us to test whether belief in conspiracy thinking is specifically linked to the level of intentionality the explanation of harm describes and whether this is associated with paranoia.

Agent presence and specificity conditions were explored in secondary analyses and we report the results of these manipulations in the electronic supplementary material. All pre-registered primary manipulations (target and intentionality) are reported in the main body of this paper.

#### 2.2.3. Perception of in-group popularity

For each item in the Components of Conspiracy Ideation Questionnaire (see electronic supplementary material), participants indicated whether people similar to them would endorse the theory (yes/no/unsure). Only answers of yes/no were included in the analyses (70% of the data: no = 4171 items (26%), yes = 6997 items (44%)). Similarity has widely been used as an in-group cue in previous research [36].

#### 2.2.4. Social and economic conservatism

We measured social and economic conservatism using the self-report Social and Economic Conservatism Scale (SECS) [37]. This scale is composed of 12 items, each corresponding to one issue (seven social, five economic), and participants are asked to rate the extent to which they feel positively or negatively towards each issue. Scores of 0 imply the greatest negativity, and scores of 100 indicate the greatest positivity. A study conducting reliability analysis on data from 319 participants confirmed the internal consistency of the scale (*α* = 0.88) [37]. This scale statistically reflects the distinguishable factors of economic and social conservatism, which mirrors a conceptual understanding in political psychology of the dissociable nature of social and economic conservatism in the US [37].

#### 2.2.5. General conspiracy mindset

We measured general conspiracy mindset using the Conspiracy Mentality Questionnaire (CMQ) [38]. The CMQ is five items long and has been shown to have cross-cultural validity in measuring general conspiracy mentality as a one-dimensional construct that is stable across time [38]. In the questionnaire, participants read five statements and rate the extent to which they agree from 0% (certainly not) to 100% (certain), on a scale with 10% intervals. In a large sample (*n* = 1640) in the English version of this questionnaire, the mean agreement per item was 6.3 (s.d. = 1.9) out of 10 (or 63%) [38]. A study investigating the test-retest reliability of the CMQ on 133 participants reported satisfactory internal consistency (*α* > 0.75 at each time point) and a correlation of 0.84 between two assessment points [38].

### 2.3. Procedure

All participants began by reporting their age and gender and then completed the R-GPTS, Components of Conspiracy Ideation Questionnaire, and positive control questionnaire (see electronic supplementary material), order randomized between participants. There were eight attention-check questions interspersed throughout these questionnaires, where 90% of participants answered all of these correctly. We re-ran all analyses excluding those who failed more than one attention check and confirmed there were no qualitative differences (see electronic supplementary material). To finish, the participants completed General Conspiracy Mindset and Social and Economic Conservatism questionnaires.

### 2.4. Primary pre-registered analyses

We used an information-theoretic (IT) approach with multi-model averaging for our regression analyses [39,40]. We ran one pre-registered cumulative link model (clm, [41]) where we standardized all continuous input variables and centred all binary input variables [42]. Endorsement of items in the Components of Conspiracy Ideation Questionnaire was the output variable, and the input variables were paranoia, target, ingroup popularity, intentionality, gender, age and interaction effects between paranoia and target, paranoia and ingroup popularity, and paranoia and intentionality. The model also included random effect terms for participant ID and theme. Paranoia refers to score on the persecutory subscale of the R-GPTS and was included as a standardized continuous input variable. The model included data for the specificity = general condition, in order to hold constant the number of items included in each target condition, as specificity was only varied in the target = society condition.

We note some variations from the pre-registered model: intentionality and an interaction term for intentionality * paranoia were included as input variables in the model given some deviation from the initial network analysis (described in the next section). Item theme was included as a random effect rather than item number as intercepts were expected to vary within each theme, and a random effect term of item number would have unintentionally controlled for variation in the main variables of interest; nationality and ethnicity were not included as an input variable as US participants only were recruited and we made no predictions pertaining to these variables.

### 2.5. Network analyses

Psychological networks are data-driven models consisting of nodes representing observed variables, where these nodes are connected by edges that represent the statistical relationships between them [43]. The edge weights depict the relationship between two nodes while controlling for all other nodes in the network. Epskamp & Fried [43] note that network analysis involves two main stages: estimating a statistical model on data and representing this as a weighted network between observed variables; and analyzing the structure of this network—for example testing significant differences between edge weights. We intended to employ network analysis to investigate the relationship between paranoia and endorsement of different types of items on the Components of Conspiracy Ideation Questionnaire, as well as whether belief in one type of conspiracy theory predicted belief in conspiracy theories with similar attributes (whether there are distinguishable ‘types’ of conspiracy thinking).

We note a deviation from our pre-registered network analysis. We initially pre-registered a network analysis where all items in the Components of Conspiracy Ideation Questionnaire would be included along with paranoia items as nodes in the network. However, the resulting network estimated had low stability, likely due to low statistical power due to the large number of items included as individual nodes, so we did not draw inferences from it as this poses problems for replicability [44,45].

Consequently, we ran an unregistered network analysis involving fewer nodes to increase power. We included one node pertaining to each type of item in the network model, where only the general conditions were included: intentional harm targeting the self (type 1), incidental harm targeting the self (type 2), intentional harm targeting society (type 3), incidental harm targeting society (type 4); as well as one node for the persecution subscale of the R-GPTS. Each participant's ratings were summed across items that corresponded to each type and converted into ordered categorical variables with four levels to be included in the network analysis. We used ordered categorical variables as distributions of these variables were skewed. Due to skew, the node reflecting persecutory ideation was also converted into an ordered categorical variable with four levels, consistent with severity thresholds identified in Freeman *et al.* [35].

This network enabled us to examine if endorsement of one type of item on the Components of Conspiracy Ideation Questionnaire was associated with endorsement of other types, in particular, testing if endorsement was most strongly associated for items specifying the same level of intentional harm or type of target. This enabled us to draw inferences pertinent to our pre-registered prediction that items specifying different levels of intentional harm and target would be more distinguishable on the basis of endorsement. The network analysis also allowed us to test how paranoia is associated with endorsement of different types of item in the Components of Conspiracy Ideation Questionnaire, where our pre-registered prediction was that paranoia would be most closely associated with type 1, followed by type 2, 3 and then 4. We could not test these predictions using the pre-registered regression analysis alone.

We estimated the network using a mixed graphical model [46], where all variables were categorical so no assumptions about distributions were made. We used absolute shrinkage and selection operator (LASSO) regularization with EBIC model selection [44] in order to provide conservative estimates and a sparse network. LASSO regularization shrinks all edge weights toward zero and sets all small weights to zero by limiting the sum of absolute parameter values. The level of penalization involved is determined by the parameter lambda, selected using Extended Bayesian Information Criterion [44]. EBIC model selection also involves a tuning parameter, gamma, which we set to 0.5 [47].

The resulting network estimated had high stability, as revealed by *case-dropping subset bootstrap* using the *bootnet* function in R [48] (see electronic supplementary material). Here we bootstrapped the model 1000 times where increasing numbers of cases are removed from the dataset and the centrality metrics (in our case Strength and Expected Influence) are recalculated with each iteration to give a correlation stability coefficient [44]. Secondly, accuracy of estimated edge-weights was calculated by a bootstrap analysis where we bootstrapped the model 500 times to construct *bootstrapped confidence intervals* (CIs), where in 95% of cases the CI contains the true value of the edge-weight parameter (see electronic supplementary material).

Next, we performed bootstrapped difference tests to explore our pre-registered prediction, that paranoia would be most closely associated with type 1 items, followed by type 2, 3 and 4 consecutively, where associations are operationalized as edge weights in the network.

As our analyses diverged from the initial network analysis pre-registered, we were not able to test whether nodes representing individual items in the Components of Conspiracy Ideation Questionnaire clustered together based on type. However, we were able to investigate whether endorsement of one type of item on the Components of Conspiracy Ideation Questionnaire was associated with endorsement of other types with similar attributes. We achieved this by examining the edge weights between nodes representing each type of item and performing bootstrapped difference tests to determine the differences in edge weights between these nodes. A weak or absent edge-weight between two nodes representing a different item type suggests a greater distinction between nodes (and hence implying a more ‘distinguishable category’), whereas stronger relationships between nodes suggest that belief in these theories is more closely related. We note that it is not possible to control for multiple testing in these significance tests [44].

We also calculated predictability estimates for each node and visualized them using the *qgraph* package in R [49]. Predictability refers to the extent to which the variance of any given node is explained by the edges connected to it: how well any given node can be predicted by neighbouring nodes in the network [50]. Predictability is an interesting metric for two principal reasons [50]. Firstly, it allows us to determine the relevance of edges connected to a node, where a node that has high predictability has more relevance in the network as it can be determined to a greater extent by surrounding nodes. Secondly, predictability is an indication of how self-determined the network is, where low predictability overall implies that the network is largely determined by variables not included in the analysis.

### 2.6. Secondary analyses

We aimed to replicate the finding reported in the existing literature that belief in one conspiracy theory is related to endorsement of others [18] by calculating the average inter-item correlation for the Components of Conspiracy Ideation Questionnaire [51]. We also tested the prediction that paranoia and GCM scores would be associated, in accordance with aforementioned literature reporting the positive correlation between paranoia and general conspiracy mindset.

We ran pre-registered exploratory analyses using the SEC and GCM data to test whether Social and Economic Conservatism and General Conspiracy Mindset were associated, as previous literature has given mixed results.

We report secondary pre-registered analyses regarding the impact of specificity, severity and recognition of items on the Components of Conspiracy Ideation Questionnaire on endorsement in the electronic supplementary material.

## 3. Results

**Figure 1.**
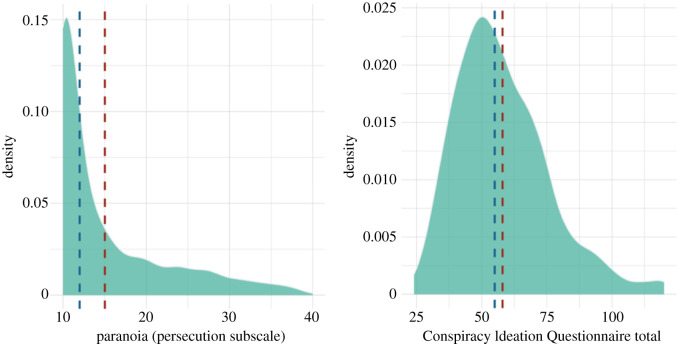
Distribution of sum scores on the persecution subscale of the R-GPTS (left panel) and the Components of Conspiracy Ideation Questionnaire (right panel). Blue dashed lines represent median scores, red dashed lines represent mean scores.

Paranoia scores spanned the full R-GPTS persecution subscale range (table 1 and figure 1). The distribution of paranoia scores was positively skewed where 37.4%, 37.2%, 16.7% and 8.7% of participants in the current study respectively fell in the *elevated*, *moderately severe*, *severe* and *very severe* categories of persecutory ideation specified by Freeman *et al*. [35]. Components of Conspiracy Ideation Questionnaire scores followed a less positively skewed distribution than paranoia scores (figure 1).

Participants who endorsed one item on our Components of Conspiracy Ideation Questionnaire were also likely to endorse others as demonstrated by the average inter-item correlation of 0.37. An inter-item correlation between 0.20 and 0.40 suggests that items are reasonably homogeneous while containing sufficiently unique variance [51]. A Mann-Whitney *U* test indicated that mean endorsement of items on the positive control questionnaire (see electronic supplementary material) was significantly higher than on the Components of Conspiracy Ideation Questionnaire (*W* *=* 854966*, p* *<* 0.001).

People scoring higher in general conspiracy mindset measured by the CMQ also were more likely to endorse items in the Components of Conspiracy Ideation Questionnaire (*r*_t_ = 0.34, *p* < 0.001). General conspiracy mindset was positively associated with paranoia (*r*_t_ = 0.21, *p* < 0.001), as predicted. General conspiracy mindset was positively associated with Social and Economic Conservatism (*r*_t_ = 0.18, *p* < 0.001).

### 3.1. Primary pre-registered analysis

Participants scoring higher in paranoia were more likely to endorse items in the Components of Conspiracy Ideation Questionnaire (estimate = 0.83, 95%CI = 0.72, 0.93; table 3), as predicted.

**Table 3.** Results of the primary pre-registered model exploring endorsement of items on the Components of Conspiracy Ideation Questionnaire (model 1). Model average estimates, unconditional standard errors, confidence intervals and relative importance for the terms included in the top model set are presented. See electronic supplementary material for details of top model set.

parameter	estimate	unconditional s.e.	95%CI
ingroup (0 = ingroup doesn't agree, 1 = ingroup does agree)	1.15	0.03	(1.08, 1.21)
intentionality (0 = incidental, 1 = intentional)	−1.51	0.04	(−1.59, −1.42)
target (0 = self, 1 = society)	0.40	0.04	(0.32, 0.48)
paranoia	0.83	0.06	(0.72, 0.93)
ingroup : paranoia	−0.16	0.03	(−0.22, −0.09)
intentionality : paranoia	0.34	0.04	(0.26, 0.42)
target : paranoia	−0.14	0.04	(−0.22, −0.06)
age	−0.01	0.03	(−0.07, 0.05)
gender	−0.004	0.05	(−0.10, 0.09)

Endorsement was stronger overall for items that described society as a whole as the target of any harm described (estimate = 0.40, 95%CI = 0.32, 0.48; table 3). As expected, those scoring high in paranoia were more likely to endorse items with self-referential targets (paranoia × target: estimate = −0.14, 95%CI = −0.22, −0.06; table 3 and figure 3).

**Figure 2.**
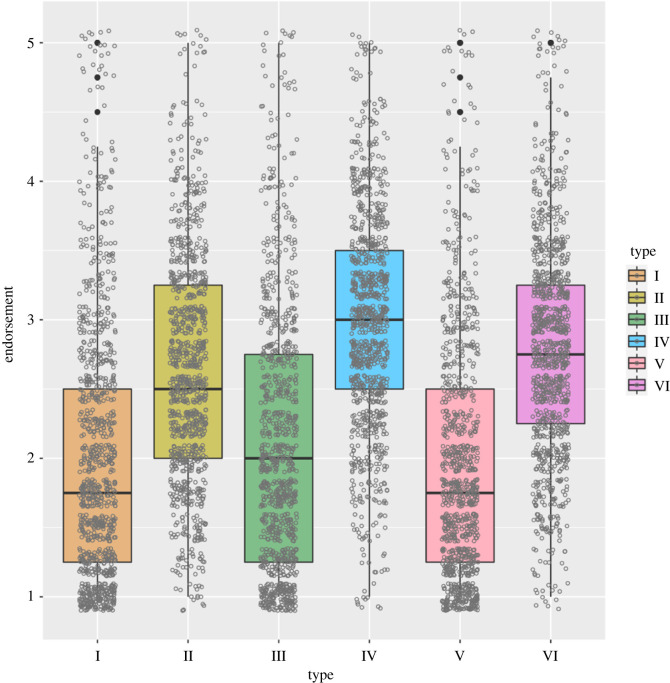
Mean endorsement of items on the Components of Conspiracy Ideation Questionnaire, according to conspiracy theory type. I = intentional, self, general; II = incidental, self, general; III = intentional, society, specific; IV = intentional, society, general; V = incidental, society, specific, VI = incidental, society, general. Mid hinges signify median endorsement values. Lower and upper hinges correspond to the 25th and 75th percentiles, and upper/lower whiskers extend from the upper/lower hinge to the largest value no greater/lower than 1.5 times the interquartile range from the hinge. Outliers beyond 1.5 times the interquartile range from the hinge are denoted as black filled points. Raw datapoints are denotes as grey circles.

**Figure 3.**
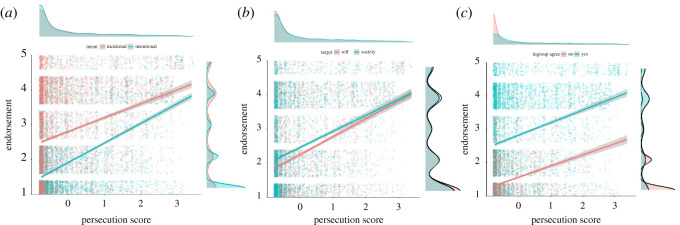
Mean endorsement of items on the Components of Conspiracy Ideation Questionnaire as a function of standardized paranoia scores on the persecutory subscale, and three separate factors: (*a*) intentionality described in the conspiracy theory, (*b*) target of the conspiracy theory and (*c*) whether the participant believes others similar to them believe in the conspiracy theory. Lines depict generalized linear model predictions. Shaded areas around each line represent 95% confidence level intervals for predictions of the generalized linear models. Distributions of standardized paranoia scores in each condition are presented above each graph, and distributions of endorsement for each condition are presented to the right of each graph.

Items describing incidental harm were more readily endorsed overall than those describing intentional harm (estimate = −1.51, 95%CI = −1.59, −1.42; table 3 and figure 2). Participants scoring high in paranoia endorsed items specifying intentional harm to a similar degree to those describing incidental harm, whereas people scoring lower in paranoia were less likely to endorse items describing intentional harm (paranoia × intentionality: estimate = 0.34, 95%CI = 0.26, 0.42; table 3 and figure 3).

Participants were more likely to endorse items in the Components of Conspiracy Ideation Questionnaire if they thought members of their in-group would too (estimate = 1.15, 95%CI = 1.08, 1.21; table 3 and figure 3). Against our expectations, the relationship between paranoia and endorsement was strongest when ingroup members were believed to endorse items (paranoia × ingroup belief: estimate = −0.16, CI: −0.22, −0.09). *Post hoc* Kruskal-Wallis rank sum test showed that those scoring higher in paranoia were more likely to report others similar to themselves as endorsing these items overall (Chi-squared = 583, *p* < 0.001, d.f. = 1).

Participants’ age and gender did not predict endorsement of items on the Components of Conspiracy Ideation Questionnaire.

Sensitivity analysis using G*Power [52] indicated that we could detect a minimum effect size of 0.01 with 80% power given our sample size of 1000.

### 3.2. Network analysis

**Figure 4.**
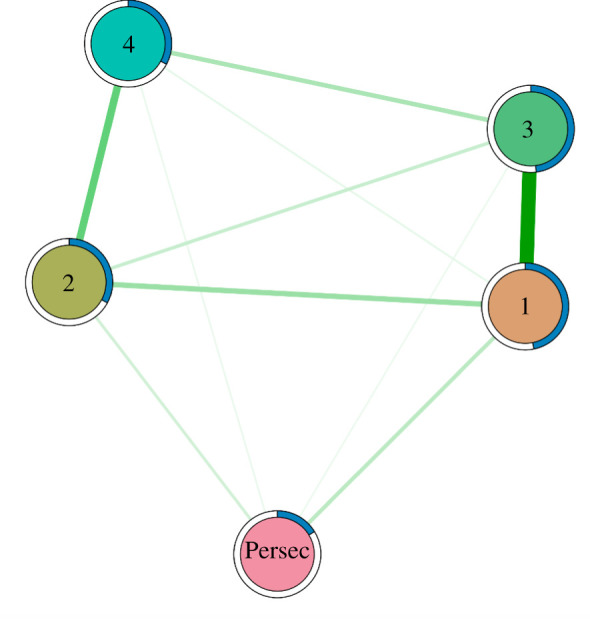
Network structure where nodes represent paranoia (Persec) and types of CT included from the Components of Conspiracy Ideation Questionnaire (1: intentional/self/general, 2: incidental/self/general, 3: intentional/society/general, 4: incidental/society/general). Edge weights are portrayed by the thickness of lines connecting nodes. Predictability of each node is represented by pie plotted on the circumference of each node.

The network structure is displayed in figure 4. Paranoia (R-GPTS persecution subscale) was significantly predicted by endorsement of all types of item in the Components of Conspiracy Ideation Questionnaire. As all nodes were included as categorical variables, and interactions between categorical variables with more than two levels are specified by more than one parameter [46], we cannot report single parameters for these relationships but rather report full parameter tables for edges connected to the paranoia node in the supplementary information.

Table 4 presents bootstrapped difference tests of edge weights between nodes included in the network. Strength of edge weights and direction of significant differences can be viewed in figure 4, where stronger edges are represented as thicker lines in the network. Bootstrapped difference tests revealed that the edge weight was stronger between paranoia and type 1 items (intentional/self/general) than paranoia and type 3 items (intentional/society/general) (CI: −0.90, −0.11). No other bootstrapped difference tests of edges joining the paranoia node reached significance, however, this was marginal in some cases: in the visualization of the network, the edge between paranoia and type 1 items was thicker than that between paranoia and type 4 items (CI: −1.07, 0.03).

Nodes representing the four different item types were interconnected. This is relevant to our first pre-registered hypothesis: conspiracy theory endorsement would be clustered along the axes of intentionality and the putative target of any harm. As discussed, although we could not perform cluster analysis on a large network including each item as an individual node in order to directly test whether the nodes clustered together on the basis of item type, our results indicate that people who endorsed items of a given type were more likely to endorse other items with similar attributes. Weak edges in the network imply that nodes are more distinguishable, and stronger edges indicate that nodes are more strongly related.

**Table 4.** Results of bootstrapped significance tests of edge weights between nodes representing different types of conspiracy theory (CT1: intentional/self/general, CT2: incidental/self/general, CT3: intentional/society/general, CT4: incidental/society/general). Colour of each table cell represents the outcome of each difference test (green = significant, orange = not significant). Statistics in each cell are the 95% confidence intervals for each difference test.

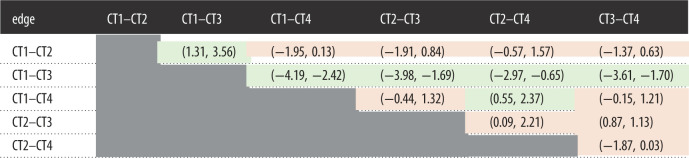

Endorsement of one item was most likely to be associated with endorsement of others that imply a similar level of intentionality. Participants who endorsed items describing intentional harm are more likely to believe in others describing intentional harm (type 1 and type 3 share a strong edge that is significantly stronger than all other edges—notably that between type 2 and 3 as well as between type 1 and 4, as these edges describe relationships between items with different levels of intentionality); and those endorsing explanations of events that describe incidental harm are also more likely to believe in others describing incidental harm (type 2 and type 4 share a strong edge, and this edge is stronger than the relationship between type 1 and type 4).

Endorsement of self-referential items was associated with endorsement of other items sharing this attribute (type 1 and type 2 share a significant edge) and this was also the case for items that describe harm targeting society (type 3 and 4 share a significant edge). These edges were weaker than those pertaining to intentionality: the edge between type 1 and type 3 nodes (both describing intentional harm with different target types) was stronger than both that between type 3 and 4 (both describing society-referential harm with different levels of intention) and that between type 2 and 1 (both describing self-referential harm with different levels of intention).

Correlation stability coefficients computed for centrality estimates were high (expected influence = 0.67, strength = 0.67; see electronic supplementary material) allowing us to be confident in the interpretations based on this network. Drawing bootstrapped CIs showed a high accuracy of edge weights in the network (see electronic supplementary material).

Predictability estimates—quantifying the extent to which any given node can be predicted by nodes that are connected to it—are represented by the pie bar surrounding each node (figure 4). The mean predictability (normalized accuracy) of all the nodes was 0.35, and type 1 and 3 items had the highest predictability (type 1: 0.48, type 2: 0.33, type 3: 0.48, type 4: 0.33, persec: 0.16).

### 3.3. Secondary analyses

The model including specificity, severity and recognition variables did not converge without errors, as such we report the results for this in our electronic supplementary material.

All results were held when re-running the models excluding participants who failed more than one attention check (see electronic supplementary material for full model results). See electronic supplementary material for models run using the reference subscale of the R-GPTS rather than the persecutory subscale.

## 4. Discussion

We present a novel study investigating the relationship between paranoia and different components of conspiracy thinking. Overall, items in our Components of Conspiracy Ideation questionnaire were endorsed to a greater extent if they described a harm that was incidental (rather than intentional), and where the outcome was more likely to affect society as a whole rather than solely the participant themselves. As expected, we found that paranoia predicted endorsement of items in this questionnaire. Paranoia was also associated with the type of item people were more likely to endorse: more paranoid individuals were more likely to endorse items describing self-referential harm, and those describing intentional harm. Both findings support our pre-registered predictions. Participants were more likely to endorse items that they thought others similar to themselves believed, but this effect was not reduced in paranoia, counter to our prediction.

A number of factors give us confidence in the generalizability of our results. Our sample had similar distribution of previous samples in general conspiracy mindset [38] and paranoia [26]. We replicate a robust finding in the literature that people who hold one conspiracy belief are more likely to also believe in others [18]. We also find that SECS was associated with conspiracy thinking, coinciding with evidence that conservatives in the United States are more likely to endorse and espouse conspiratorial theories and world views ([13]; but see [14]). We note that the data were collected during the coronavirus pandemic and, therefore, paranoia may be higher compared to normal times [12]; however this variable followed a positively skewed distribution as identified in previous research [35].

The network analysis indicated that belief in one item on our Components of Conspiracy Ideation Questionnaire was associated with belief in others, but that this varied according to the features of the conspiracy theories described. Edges were particularly strong between nodes representing item types that captured a similar level of intentionality or that specified the same target of the harm described (society/self). This clustering of endorsement for conspiracy theories items appeared to be stronger along the intentionality axis than along the target of harm axis. Supporting the interpretation that endorsement of conspiracy theories is differentiable according to these features, the edge between the most distinct items (type 1—type 4) was the weakest between nodes.

Most people endorsed items on the Components of Conspiracy Ideation Questionnaire describing incidental harm to a greater extent than those describing intentional harm. This general reluctance to attribute harmful intentions to others has been found in multiple studies. Specifically, in live interactions with others, participants are more likely to rationalize being treated unfairly as due to the self-interest of other players, rather than their harmful intent [25–27,53].

Regression analysis revealed that more paranoid individuals more strongly endorsed items on the Components of Conspiracy Ideation Questionnaire overall—and did not endorse incidental harm items to a greater extent than intentional harm items, unlike those scoring lower in paranoia. Together these findings suggest that increasing paranoia was associated with an increased tendency to believe conspiracy theories that suggest that harmful outcomes are intended. This result directly relates to the most common characteristic of paranoia: the belief that others *intend* harm, where recent research shows that paranoia is associated with an increased perception of intentionality for negative events when they occur [28]. Indeed, experimental studies have also found that more paranoid individuals make stronger attributions of harmful intent [25–27,53]. Our results extend this research: more paranoid individuals are more likely to endorse conspiracy theories that imply that the perceived harm was intended.

Participants were generally more likely to endorse items on the Components of Conspiracy Ideation Questionnaire that specified society as a whole as the target of the harm described, rather than the believer alone. It may be that personally relevant items were scrutinized to a greater degree by the participants in the current study, who consequently found them less convincing—as personally relevant messages have been shown to be processed in more depth (e.g. [54]). As expected, the primary pre-registered regression analysis found that more paranoid individuals were more likely to endorse self-referential items, although this effect was marginal. Bootstrapped difference tests of our estimated network supported this result as the edge between paranoia and self-referential items describing intentional harm (type 1) was stronger than that between paranoia and society-referential items describing intentional harm (type 3).

Participants were more likely to report that people similar to them endorsed items in the Components of Conspiracy Ideation Questionnaire that they endorsed themselves, in line with our predictions. This strong ingroup effect we found coincides with the large body of literature documenting the influence of group membership on behaviour and attitudes. Recent research highlights that the role of social influence is particularly strong with respect to conspiracy thinking [30]. Our results cannot speak to a causal relationship, but rather indicate that people who endorse conspiracy theories are likely to report that others similar to them do so too. We expected that participants scoring higher in paranoia would be less likely to endorse conspiracy theories that are perceived to be popular by members of their ingroup, as paranoia has previously been associated with social disconnection [31,55]. Counter to these expectations, however, more paranoid individuals were more likely to believe that others similar to them would also endorse items they endorsed. We note that this effect was marginal and warrants replication. However, if the effect is replicated, it may be that individuals who score higher in paranoia have smaller social networks in general, but affiliate more strongly to the few ingroup members they do have. This would mirror evidence that conspiracy communities are often marginalized and have high commitment to their ingroup [56]. It may also be the case that individuals high in paranoia are less accurate in judging social consensus, potentially leading to high levels of illusory consensus in beliefs [57]—something that also needs further investigation. It is also possible that paranoia might only reduce the conviction that others share the belief in conspiracy theory at more severe levels. Patients diagnosed with schizophrenia are often well aware that others don't share their delusional beliefs [58]. However, the extent to which this is a result of interaction with the mental health system (where highlighting this discrepancy may be an explicit part of assessment or treatment) or severity of paranoia remains to be investigated.

We note that the questionnaire design of the study was not designed to capture the full extent of participants' conspiracy beliefs. In order to isolate the variables of interest with the best level of control possible, we used prescriptive items in the Components of Conspiracy Ideation Questionnaire. As such, we did not measure a vast number of possible conspiracy beliefs—indeed the questionnaire largely focused on those involving government powers. Future research could investigate whether our results hold when applied to a broader range of conspiracy beliefs, for example by eliciting them from the participants themselves rather than asking participants to rate their endorsement of beliefs provided by the experimenter. Additionally, we note that the conspiracy theories we included that specify incidental harm do not necessarily fit with the definition of conspiracy beliefs that imply intentional harm by a group of actors—for example we note they did not all state that authorities attempted to cover-up the harms stated. However, as we have discussed in the introduction, many modern-day conspiracy theories do vary in the degree to which they imply intentional action, therefore our results can speak to how this variation relates to how convincing any given explanation of a harmful event may be. Future research might investigate endorsement of conspiracy theories along a continuum of intentionality, for example where the level of intent present in each conspiracy theory is rated by a separate panel of participants, to replicate and extend the current study.

Our results may have wider implications for research concerning belief updating. That is, as our results suggest that the level of intentional harm and the type of target conspiracy theories describe may influence the traction they receive, it is possible that the erratic belief updating processes associated with conspiracy thinking [12] may vary depending on these features of the conspiracy beliefs; further, paranoia may differentially impact individual's abilities to update their conspiracy beliefs, based on these features of such beliefs. Further research is needed to explore such possibilities.

Overall, we show that the believability of conspiracy theories may depend on the level of intentional harm implied, and who is specified as the target of the harm described. Items in our Components of Conspiracy Ideation Questionnaire that describe incidental harm, and harm that targets society as a whole, were endorsed more strongly. Endorsement of any given item was particularly associated with endorsement of other items that specified similar levels of intentionality. Pre-registered regression analysis revealed that individuals scoring high in paranoia were more likely to endorse items in this conspiracy ideation questionnaire overall, and that people scoring higher in paranoia are more likely to endorse theories describing intentional harm and those that target the believer themselves. Network analysis partially replicated these results, for example indicating that belief in self-referential items describing intentional harm is more closely associated with paranoia than belief in these items that describe harm that targets society as a whole. Participants were more likely to endorse conspiracy-type beliefs that they thought would be supported by their ingroup members, and this effect increased with paranoia. As such, our results speak to a number of unanswered questions on how paranoia relates to the components of conspiracy thinking; as well as how the features of conspiracy theories relate to how believable they are overall.

## Data accessibility

Full materials, data and code are available at https://osf.io/zx8me/?view_only=d02e5abdf6304fb0885ccf32853934ca. The study design, sample size, exclusion criteria and analyses were pre-registered at https://aspredicted.org/blind.php?x=wa2jh4.
